# Treating an Advanced Combined Hepatocellular-Cholangiocarcinoma with a Multikinase Inhibitor

**DOI:** 10.7759/cureus.53394

**Published:** 2024-02-01

**Authors:** Teresa Fraga, Nuno Bonito

**Affiliations:** 1 Medical Oncology, Instituto Português de Oncologia de Coimbra Francisco Gentil, Coimbra, PRT

**Keywords:** rare malignancy, hepatocarcinoma, treatment choices, multikinase inhibitor, combined hepatocellular-cholangiocarcinoma

## Abstract

Combined hepatocellular-cholangiocarcinoma (cHCC-CC) is an aggressive hepatic cancer that has characteristics of both hepatocellular carcinoma (HCC) and cholangiocarcinoma (CC). For resectable disease, liver resection is the preferred first treatment option. As for the advanced or metastatic setting, and due to its rarity, there is still no consensus on which is the optimal systemic treatment. As such, regimens used in both HCC and CC have often been used as first-line treatment options. We report a case of a male patient in his 50s, diagnosed with a cHCC-CC with lymph node and adrenal metastasis, with an extensive portal vein tumour thrombosis, that started treatment with a multikinase inhibitor - lenvatinib.

## Introduction

The definition of a combined hepatocellular-cholangiocarcinoma (cHCC-CC) has evolved over the years with the latest definition from the World Health Organization in 2019 describing this rare liver malignancy as a display of biopsy of histomorphological and molecular characteristics of hepatocellular carcinoma (HCC) and cholangiocarcinoma (CC) [1,2]. In Western countries, cHCC-CC comprises 1-5% of all primary liver cancers [1,3]. When deemed feasible, liver surgery with lymph node dissection is considered the standard of care for resectable cases [2]. Nevertheless, even after surgical resection, tumor recurrence remains high (almost 80% at five years) and the five-year survival rates do not exceed 30% [1]. Furthermore, most patients are often diagnosed at an advanced stage, where surgery is no longer a feasible option [1]. Since optimal management of advanced or metastatic cHCC-CC is still unclear, clinicians frequently try to define the dominant phenotype based on radiologic characteristics and the existence of tumor markers like alfa-fetoprotein (AFP) and carbohydrate antigen 19-9 (CA 19-9) and recommend standard of care for either HCC or CC [1]. If possible, molecular analysis of the tumor should be carried out to identify potentially actionable targets [1].

## Case presentation

**Figure 1:**
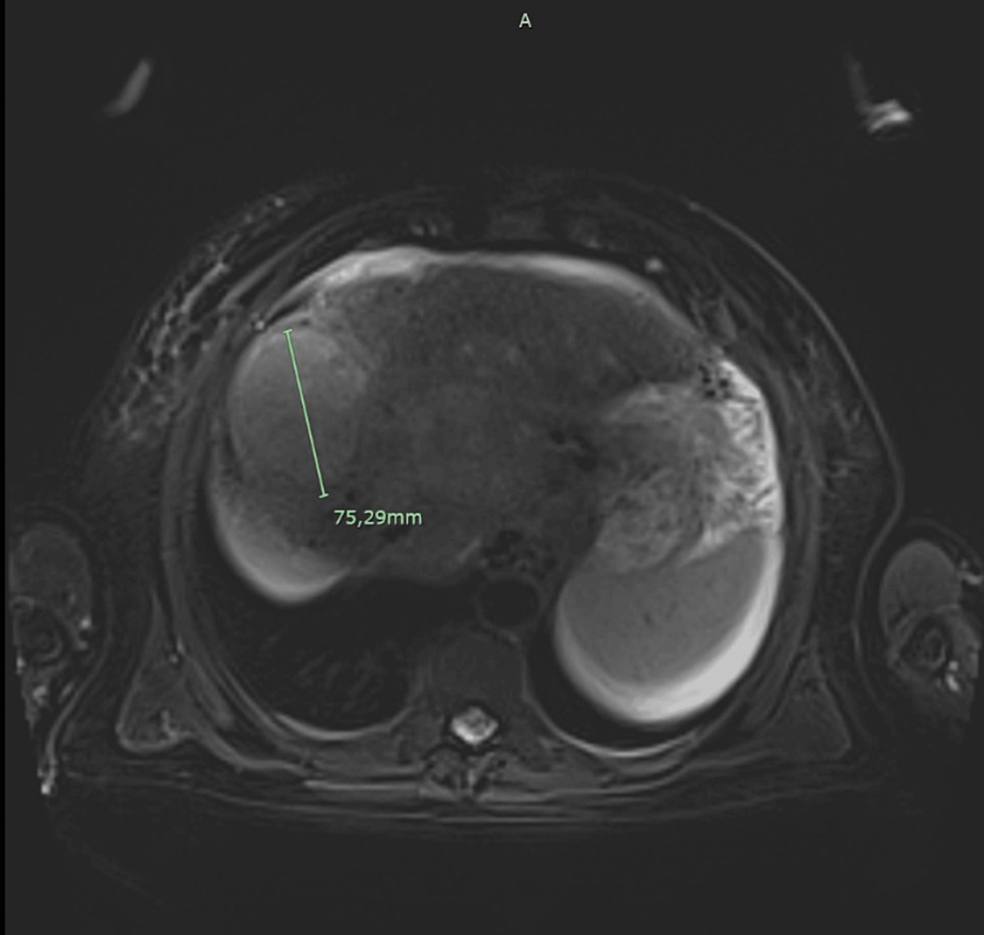
Initial MRI

We present the case of a 55-year-old male patient, that was referred to our oncology centre for systemic treatment after the diagnosis of a cHCC-CC. The patient had an Eastern Cooperative Oncology Group - Performance Status Scale of 0 (ECOG-PS), and a past medical history of liver cirrhosis of multifactorial etiology (alcohol, hereditary hemochromatosis and metabolic syndrome), with portal hypertension, Child-Pugh B (7 points) and a Model for End-Stage Liver Disease (MELD) score of 12. Approximately four months before the referral, there was only one nodular hepatic lesion (segment VIII) with 45x53mm, and at this time the patient was submitted to a transarterial radioembolization, with no response (tumor growth). When the patient was admitted to our centre, we performed a hepatic magnetic resonance imaging (MRI) on March 2023, which described multiple liver nodules, the largest with 70x60mm, with metastasis on the left adrenal gland, multiple perihepatic and lateral aortic lymph node metastasis, and extensive tumor thrombosis of the portal vein that extended until the splenoportal confluence (Barcelona Clinic Liver Cancer classification (BCLC) C) (Figure 1). This patient was deemed unsuitable for chemotherapy given his hematological alterations (thrombocytopenia). The tumor markers AFP and CA 19.9 were both elevated, with an AFP of 919 IU/mL (normal range ≤5.8 UI/mL) and CA 19.9 of 190 IU/mL (normal range ≤37 IU/mL), before treatment. We also requested a molecular somatic study of the tumor in search of fibroblast growth factor receptor (FGFR), neurotrophic receptor tyrosine kinase (NTRK) and isocitrate dehydrogenase (IDH) mutations, in order to identify potentially targetable mutations, however, no mutations were identified.

With histology of a cHCC-CC BCLC stage C, the patient was discussed in our multidisciplinary team meeting and proposed for systemic treatment. Given that our patient was not fit for chemotherapy, and based on the recent results from the HIMALAYA trial (in hepatocellular cancer) and TOPAZ-1 trial (in CC), in which durvalumab seemed to have an effect on both of these malignancies, we requested an authorization from our Pharmacy and Therapeutics Committee to use this drug. Unfortunately, our request was denied, and we decided to start treatment, based on the first-line options for HCC, with a multikinase inhibitor - lenvatinib. After starting treatment (lenvatinib 12mg id) there was a marked improvement in the tumor markers with a reduction of AFP from the initial 919 to 137 IU/mL and also of the CA 19-9 from 190 to 169 IU/mL after only one month on lenvatinib.

**Figure 2:**
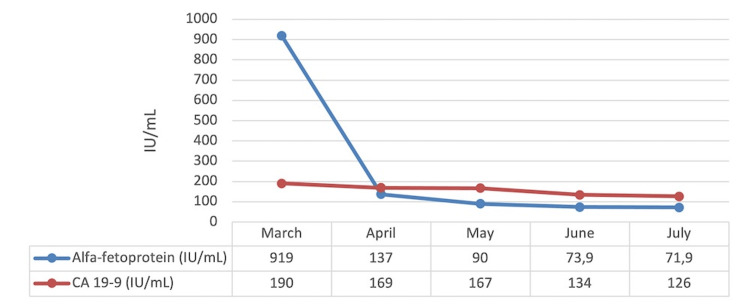
Tumour markers evolution CA 19-9: carbohydrate antigen 19-9

**Figure 3:**
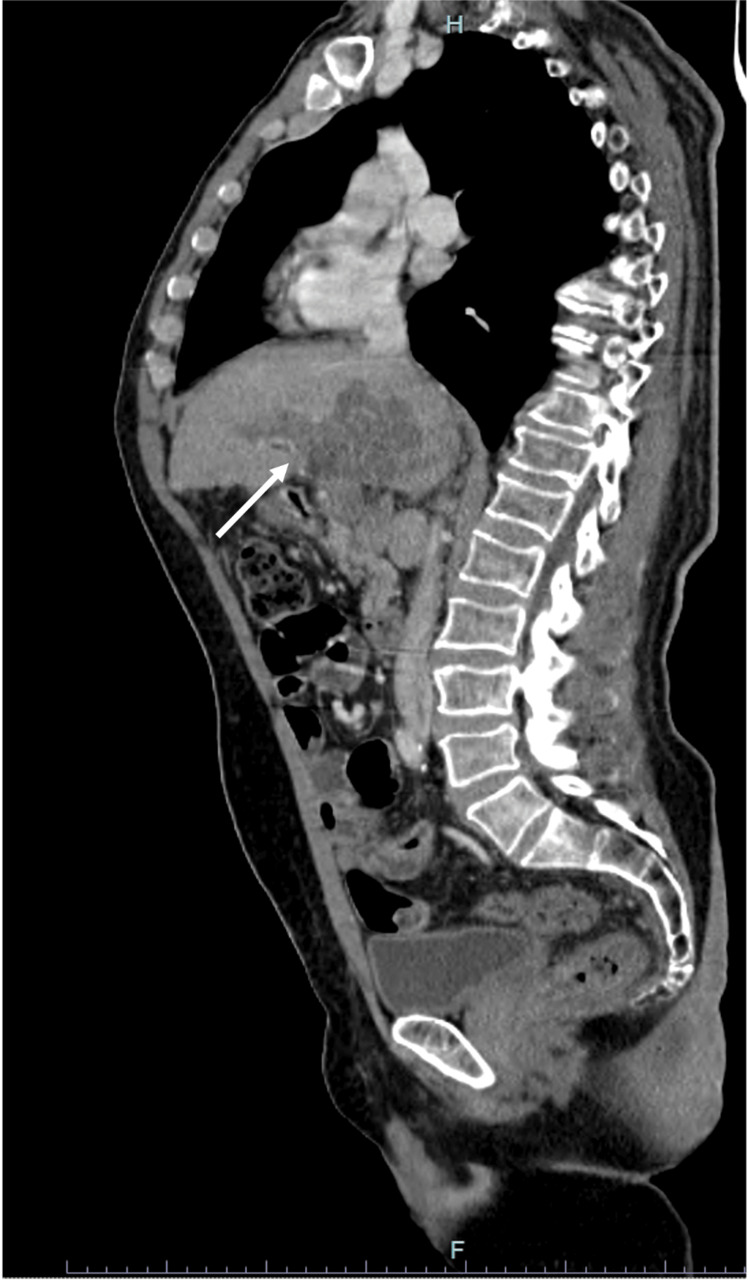
MRI three months after lenvatinib (stable disease)

After two months, the patient was admitted to the gastroenterology department due to upper gastrointestinal bleeding due to esophageal varices rupture - having undergone ligation. An attempt was made in order to place a transjugular intrahepatic portosystemic shunt (TIPS) but this was not possible due to the extensive portal thrombosis. After this episode, about a month later, the patient re-started lenvatinib with a dose reduction from 12 to 8mg id. At the first evaluation of response, after three months, tumor markers continued to decrease (AFP of 71 IU/mL), with the CT scan showing a stable disease (Figures 2-3).

Although there was disease stability, with the continued biochemical response, due to the patient’s other comorbidities, he died almost a year after diagnosis, and after seven months on lenvatinib.

## Discussion

cHCC-CC represents a rare type of liver cancer, accounting for less than 5% of all primary liver tumors [1,4]. This malignancy seems to be more common in men, with a mean age at diagnosis of around 64 years old [1,5,6]. The true prevalence of this tumor is probably underestimated due to the absence of standard confirmation of HCC diagnosis by biopsy in cirrhotic patients even though cirrhosis is commonly found in cHCC-CC, in almost 25-55% of patients [1,6]. With our patient the diagnosis was made at an earlier age (55 years old) than what is described in current literature and cirrhosis was indeed present.

The diagnosis of cHCC-CC is based on routine histological diagnosis with hematoxylin-eosin (H&E) staining, and requires the unequivocal exhibition of both cholangiocytic and hepatocytic differentiation in the same tumor [5,6]. Risk factors for cHCC-CC are comparable to those of other primary liver cancers, such as metabolic syndrome, excessive alcohol intake, liver cirrhosis and chronic viral infections (hepatitis B can C) [1,6]. This was also the case with our patient, who had a heavy past medical history of liver cirrhosis for many years due to a combination of three entities: metabolic syndrome, excessive alcohol and hereditary hemochromatosis. In our case, we had a biopsy that made the diagnosis.

Recently, Pomej et al. published a report from their European multicentre retrospective study that analyzed 101 patients with the diagnosis of cHCC-CC, where they report that although the majority of patients had a preserved liver function (Child-Pugh A) they were also mostly diagnosed at an advanced stage (BCLC B-D) [5]. As such, only a minority of patients will be candidates for surgery, that is, when feasible, the best therapeutical option. Nevertheless, even after surgery, the risk of recurrence has been reported to be higher than for HCC (70-80% at five years) and five-year survival rates do not exceed 30% [1,3,6]. The treatment sequence in this advanced setting (recurrent), as well as for metastatic disease, is still not optimized, and follows mostly the existing guidelines for CC or HCC [1,3,4].

For patients with locally advanced or metastatic CC, the standard first-line chemotherapy is the combination of gemcitabine and cisplatin, yielding a median overall survival (mOS) of 11.7 months [1]. In the TOPAZ-1 trial, the addition of the programmed cell death ligand 1 (PD-L1) antibody durvalumab to the standard of care chemotherapy resulted in an improvement in mOS (11.5 to 12.8 months) with more than twice as many patients alive at 24 months (24.9% versus 10.4%), as well as improved progression-free survival (PFS) and overall response rate (ORR) [1,7]. In the HIMALAYA trial (on HCC) durvalumab monotherapy proved to be non-inferior to sorafenib for patients with unresectable HCC (mOS of 16.6 months, with OS at 36 months of 24.7%) [8]. Based on the results from these two trials, and since chemotherapy was contraindicated in our patient, durvalumab was one of the options we considered for the treatment of this patient. However, this option was denied by our Pharmacy and Therapeutics Committee.

For many years the multikinase inhibitor sorafenib stood as the established first-line treatment for advanced HCC, with a mOS of 10 to 12 months [1,9]. In 2018, the phase III REFLECT trial established lenvatinib as a non-inferior alternative to first-line sorafenib, with superior tolerability, a higher objective response rate, and delayed tumor progression [1,10].

Since there are no prospective trials, treatment options are based on reports from retrospective observational studies and case reports [1]. Gigante et al. conducted a retrospective analysis with a cohort of 83 patients with unresectable/metastatic cHCC-CC who were treated with tyrosine kinase inhibitor (TKI) or with systemic chemotherapy [1,6]. In this work, and contrary to previous reports where platinum-containing regimens were associated with more favorable outcomes, it seemed that first-line systemic treatments with TKIs have a similar efficacy to platinum-based chemotherapies, in patients with advanced cHCC-CC [1,6]. Also, in the report from Zhao et al. where they review the different locoregional or medical treatment options for advanced cHCC-CC, they conclude that TKI (either sorafenib or lenvatinib) is a therapeutic alternative, especially when patients are not fit for chemotherapy [1]. Since our first potential treatment option was not approved, and given that the patient’s comorbidities made him unfit for chemotherapy, lenvatinib was chosen as our first-line option. This option resulted in a continued biochemical response, with a decrease in both tumor markers (particularly the AFP value), and a stable disease at the first evaluation of response.

Loosen et al. also reported a possible activity of lenvatinib in a cHCC-CC but as a third-line therapy, which resulted in a mixed response after three months of treatment (partial response on brain metastasis but progression on bone metastasis) [1,11]. In the first-line setting, Osuga et al. also reported a case of a male patient, 77-years-old, diagnosed with a cHCC-CC and treated with lenvatinib (8mg id), with a maintained response for at least seven months [1,3].

## Conclusions

The treatment of a cHCC-CC poses a significant challenge due to the absence of established guidelines. To the best of our knowledge, our case is only the second to report the use of lenvatinib as a first-line treatment in this advanced setting of this rare entity. This case further exemplifies the complexities associated with managing patients with liver cirrhosis and emphasizes the need for an individualized and tailored therapeutic approach. While acknowledging the limitations of a single-case report, it offers valuable insights and highlights the need for further research to refine treatment strategies for this rare and complex malignancy.
